# *Trypanosoma brucei* Inhibition by Essential Oils from Medicinal and Aromatic Plants Traditionally Used in Cameroon (*Azadirachta indica*, *Aframomum melegueta*, *Aframomum daniellii*, *Clausena anisata*, *Dichrostachys cinerea* and *Echinops giganteus*)

**DOI:** 10.3390/ijerph14070737

**Published:** 2017-07-06

**Authors:** Stephane L. Ngahang Kamte, Farahnaz Ranjbarian, Gustavo Daniel Campagnaro, Prosper C. Biapa Nya, Hélène Mbuntcha, Verlaine Woguem, Hilaire Macaire Womeni, Léon Azefack Tapondjou, Cristiano Giordani, Luciano Barboni, Giovanni Benelli, Loredana Cappellacci, Anders Hofer, Riccardo Petrelli, Filippo Maggi

**Affiliations:** 1 School of Pharmacy, University of Camerino, 62032 Camerino, Italy; landry.ngahangkamte@unicam.it (S.L.N.K.); loredana.cappellacci@unicam.it (L.C.); filippo.maggi@unicam.it (F.M.); 2 Department of Medical Biochemistry and Biophysics, Umeå University, 90187 Umeå, Sweden; farahnaz.ranjbarian@umu.se (F.R.); anders.hofer@umu.se (A.H.); 3 Institute of Infection, Immunity and Inflammation, College of Medical, Veterinary and Life Sciences, University of Glasgow, Glasgow G12 8TA, UK; g.campagnaro.1@research.gla.ac.uk; 4 Laboratory of Medicinal Plant Biochemistry, Food Science and Nutrition, Department of Biochemistry, Faculty of Sciences, University of Dschang, Dschang POX 67, Cameroon; prbiapa@yahoo.fr (P.C.B.N.); helenembuntcha@yahoo.fr (H.M.); woverly91@yahoo.fr (V.W.); womeni@yahoo.fr (H.M.W.); 5 Laboratory of Environmental and Applied Chemistry, Faculty of Science, University of Dschang, Dschang POX 67, Cameroon; tapondjou2001@yahoo.fr; 6 Instituto de Física, Universidad de Antioquia, Medellín AA 1226, Colombia; cristiano.giordani@udea.edu.co; 7 School of Science and Technology, Chemistry Division, University of Camerino, 62032 Camerino, Italy; luciano.barboni@unicam.it; 8 Department of Agriculture, Food and Environment, University of Pisa, 56124 Pisa, Italy; benelli.giovanni@gmail.com

**Keywords:** essential oils, African trypanosomiases, *Trypanosma brucei*, Cameroon, aromatic and medicinal plants

## Abstract

Essential oils are complex mixtures of volatile components produced by the plant secondary metabolism and consist mainly of monoterpenes and sesquiterpenes and, to a minor extent, of aromatic and aliphatic compounds. They are exploited in several fields such as perfumery, food, pharmaceutics, and cosmetics. Essential oils have long-standing uses in the treatment of infectious diseases and parasitosis in humans and animals. In this regard, their therapeutic potential against human African trypanosomiasis (HAT) has not been fully explored. In the present work, we have selected six medicinal and aromatic plants (*Azadirachta indica*, *Aframomum melegueta*, *Aframomum daniellii*, *Clausena anisata*, *Dichrostachys cinerea*, and *Echinops giganteus*) traditionally used in Cameroon to treat several disorders, including infections and parasitic diseases, and evaluated the activity of their essential oils against *Trypanosma brucei* TC221. Their selectivity was also determined with Balb/3T3 (mouse embryonic fibroblast cell line) cells as a reference. The results showed that the essential oils from *A. indica*, *A. daniellii*, and *E. giganteus* were the most active ones, with half maximal inhibitory concentration (IC_50_) values of 15.21, 7.65, and 10.50 µg/mL, respectively. These essential oils were characterized by different chemical compounds such as sesquiterpene hydrocarbons, monoterpene hydrocarbons, and oxygenated sesquiterpenes. Some of their main components were assayed as well on *T. brucei* TC221, and their effects were linked to those of essential oils.

## 1. Introduction

For centuries, people living in Africa have been facing various infectious tropical illnesses, among which African trypanosomiases are some of the most frequent relevant parasitic diseases. African trypanosomiases, commonly called sleeping sickness in humans (HAT; Human African Trypanosomiasis) and Nagana in domestic livestock, affect a huge number of people living in poverty in 36 sub-Saharan countries and hence have a key socioeconomic impact [1,2,3]. After a century of outbreaks, due to political instability and a lack of funding, around 70 million people and 50 million cattle are still at risk of exposure in Africa [4].

Trypanosomiasis is transmitted by the bite of insects from the *Glossina* spp. (Glossinidae) and is fatal in humans, if untreated. While taking a blood meal, infected *Glossina* flies can spread extracellular protozoans from the species *Trypanosoma brucei.* There are three morphologically indistinguishable subspecies of *T. brucei*. The subspecies *T. b. gambiense* is responsible for a chronic form of the human disease, while *T. b. rhodesiense* causes an acute form, which more rapidly leads to death. Both subspecies are infective to humans, whereas *T. b. brucei* is only infective to animals. During the early stage of the disease or the hemolymphatic phase, the parasite is restricted to the blood and lymph, and, after months or years, it invades the central nervous system, resulting in various neurological symptoms, including sleeping disturbance [3].

As for other neglected tropical diseases, the chemotherapeutical arsenal against HAT is based on limited, expensive, and often toxic medicines that are administered parentally in a context of poverty and a lack of qualified personnel in healthcare centers. The few drugs that are available are pentamidine and suramin for the early stage of the disease and eflornithine (also in combination with nifurtimox) and melarsoprol, an organoarsenic compound, for the late stage, when the parasite infects the brain. Although melarsoprol can cause severe reactive encephalopathy, it remains a first line treatment for infections by *T. b. gambiense* in many rural areas because of the high cost of eflornithine [1]. Overall, the scenario described above highlights the critical nature of the current situation and the urgent need to explore new sources of potentially effective and safe compounds for therapy.

It has been estimated that a large part of the African population relies on herbal medicines as the first-line treatment for different ailments. In this regard, the African flora represents a valuable source of anti-infectious compounds to be exploited as drugs [5,6,7]. In the fight against *Trypanosoma* infections, new therapeutic options can be provided by plant extracts, essential oils, and plant-borne compounds [6,8,9,10]. Essential oils are volatile mixtures distilled from aromatic plants and composed of several dozens of components such as terpenoids, phenylpropanoids, and aliphatic compounds [11,12]. In recent years, an increasing interest on essential oils as alternative/integrative therapies in the treatment of HAT has been observed [13]. Essential oils and representative components from lemongrass (*Cymbopogon citratus* (DC.) Stapf), oregano (*Origanum vulgare* L.), thyme (*Thymus vulgaris* L.), clove (*Syzygium aromaticum* (L.) Merr. and L.M. Perry), basil (*Ocimum basilicum* L.), and yarrow (*Achillea millefolium* L.) exhibited efficacy both in in vitro and in vivo models of trypanosomal infections [14]. Essential oils are composed of a plethora of chemical compounds, which have various modes of action on microorganisms, and, additionally, essential oils do not induce any form of resistance [15].

In the present study, we selected a panel of Cameroonian medicinal and aromatic plants as potential sources of anti-trypanocidal compounds. We focused on *Azadirachta indica* A. Juss (Meliaceae), *Aframomum melegueta* K. Schum. (Zingiberaceae), *Aframomum daniellii* (Hook. F.) K. Schum. (Zingiberaceae), *Clausena anisata* (Willd.) Hook.f. ex Benth. (Rutaceae), *Dichrostachys cinerea* (L.) Wight and Arn. (Mimosaceae), and *Echinops giganteus* A. Rich. (Asteraceae).

*A. indica*, also known as ‘neem tree‘, is considered by many people living in Africa as a miraculous plant for a wide range of uses in ethnopharmacology such as anthelmintic, antimalarial, anti-inflammatory purposes and for healing skin diseases. Most of these properties were then confirmed by scientific reports [16,17,18]. All parts of the plant can be used for medicinal purposes, including the seed oil extracted by mechanical pressure [19,20]. Notably, the ethanolic extract obtained from neem stem bark exhibited activity against *T. b. brucei* [21], whereas the leaf essential oil has been barely investigated to date.

*A. melegueta,* also known as ‘alligator pepper’ or ‘grain of paradise’, is a perennial plant native to western Africa, and its seeds are used as a spice in food due to their aromatic flavor and pungent taste or as ingredients of ethnomedical preparations for the treatment of snakebites, stomachaches, and diarrhea [22]. Antimicrobial, anti-inflammatory, anticancer, and antioxidant properties have been reported for alligator pepper [23]. Concerning seed volatile constituents, they showed repellent activity against adults of the maize weevil *Sitophilus zeamais* [24].

*A. daniellii*, also known as ‘African cardamom’, is an herbaceous plant traditionally used in Africa as a spice due to the pungent taste of its seeds, whereas, for medicinal purposes, the plant is employed as a laxative and for curing parasitic and other microbial infections [25,26]. The anti-inflammatory effect of its seed essential oil and the preservative properties in stored grains have also been reported [27,28].

*C. anisata* is an evergreen tropical tree up to 10 m tall with leaves containing secretory glands and emitting a strong smell [29]. In Africa, it is considered highly effective against insects and has also been used in the treatment of malaria [30].

*D. cinerea* is a tree growing in tropical areas in countries such as Cameroon, Kenya, South Africa, and Tanzania, where a decoction of its leaves and roots is used against venereal disease, eye inflammations, skin diseases, and snake bites. The root is used for chest complaints and the twigs for gonorrhoea and syphilis. The essential oil was toxic to mosquito vectors of bancroftian filariasis [26].

*E. giganteus* is a perennial herb widely used in African traditional medicine for the treatment of various ailments [31,32]. In previous studies, the root methanolic extract showed significant antibacterial [33], antifungal [34], and antioxidant effects [35]. The cytotoxicity of the crude methanol extract from the roots has also been demonstrated [36,37].

Overall, these Cameroonian plants are also traditionally used to control populations of arthropod pests [38,39,40]. In this research, we shed light on the growth inhibitory potential of the essential oils obtained from the leaves of *A. indica*, *A. daniellii*, and *C. anisata*; the seeds of *A. melegueta* and *D. cinerea*; and the roots of *E. giganteus* against *T. brucei* TC221. Selected pure constituents from the above mentioned essential oils were also evaluated.

## 2. Materials and Methods

### 2.1. Plant Material

Leaves of *A. indica* were collected during the dry season (January 2016) from a tree in the city of Guidiguis (north of Cameroon), about 70 km from Maroua. The leaves were air-dried in the shade for one week and kept in papers. Fruits (pods) of *A. melegueta* were collected in a forest near Foumbam (western Cameroon) in December 2015. Once harvested, the seeds were removed from their pods and dried at room temperature over a period of three weeks in the absence of sunlight. At the end of the drying process, the seeds were placed into paper bags before hydrodistillation. Leaves of *C. anisata* were collected in the village of Baffou, Menoua Division, Western Cameroon. Leaves of *A. danielli* and fruits of *D. cinerea* and roots of *E. giganteus* were collected from Bamougoum and Bafoussam’s market (Cameroon, Western Region), respectively. The pericarp of *D. cinerea* was removed and the seeds used; the roots of *E. giganteus* were washed with water and sliced into small pieces. These plant parts were dried at room temperature for one week. The botanical identification of the five species was performed by a taxonomist at the Cameroon National Herbarium (Yaoundé, Cameroon), and the voucher specimens were archived with the following codes: 4447 SRFK (*A. indica*), 43117 HNC (*A. melegueta*), 43130 HNC (*A. danielli*), 44242/HNC (*C. anisata*), 42920 HNC (*D. cinerea*), and 23647 SRF (*E. giganteus*). The botanical names were also checked against The Plant List database (www.theplantlist.org).

### 2.2. Isolation of Essential Oil

The dry leaves of *A. indica, A. daniellii*, and *C*. *anisata*; the seeds of *A. melegueta* and *D. cinerea*; and the roots of *E. giganteus* were cut into small pieces and subjected to hydrodistillation using a Clevenger-type apparatus until no more oil was obtained. The essential oils obtained were dried using Na_2_SO_4_ and stored at −20 °C in vials sealed with teflon caps and protected from light before use. The oil yields were calculated on a dry weight basis (%, *w/w*).

### 2.3. Chemicals

For the identification of volatiles, the following analytical standards purchased from Sigma Aldrich (Milan, Italy) were used: α-pinene, β-pinene, sabinene, 1,8-cineole, camphene, myrcene, α-phellandrene, δ-3-carene, *p*-cymene, limonene, γ-terpinene, terpinolene, linalool, *trans*-pinocarveol, terpinen-4-ol, α-terpineol, myrtenal, citronellol, isobornyl acetate, (*E*)-caryophyllene, α-humulene, (*E*)-β-ionone, and caryophyllene oxide. The reference drug suramin was purchased from Sigma Aldrich.

### 2.4. Gas Chromatography–Mass Spectrometry (GC-MS) Analysis of Essential Oils

The chemical constituents of the Cameroonian essential oils were analyzed on an Agilent 6890 N gas chromatograph coupled to a 5973 N mass spectrometer (Santa Clara, CA, USA) and equipped with a HP-5 MS capillary column (5% phenylmethylpolysiloxane, 30 m, 0.25 mm i.d., 0.1 μm film thickness; J & W Scientific, Folsom, CA, USA). For the separation of the volatile constituents, the following temperature program was used: 5 min at 60 °C then 4 °C/min up to 220 °C, then 11 °C/min up to 280 °C held for 15 min. The injector and detector temperatures were: 280 °C; carrier gas: Helium; flow rate: 1 mL/min; split ratio: 1:50; acquisition mass range: 29–400 *m/z*; and mode: electron-impact (EI, 70 eV). The essential oil was diluted 1:100 in *n*-hexane, and then 2 µL of the solution was injected into the GC-MS system. For the identification of essential oil components, co-injection with the authentic standards available in our laboratory (purchased from Sigma-Aldrich) was performed, together with a comparison of the retention indices and the mass spectra of those occurring in the ADAMS, NIST 08, and FFNSC2 libraries [41,42,43]. The percentage values of the volatile components were the means of three chromatographic analyses and were determined from the peak areas without the use of correction factors. 

### 2.5. T. brucei and Mammalian Cell Culture and Growth Inhibition Assay

The cell culture conditions and the growth inhibition assay on *T. brucei* and Balb/3T3 cells were performed as described before [44]. *T. brucei* TC221 bloodstream forms and mouse embryonic fibroblast Balb/3T3 cells (ATCC no CCL-163) were cultivated in vented plastic flasks at 37 °C with 5% CO_2_. For *T. brucei*, the growth medium was HMI-9 [45] supplemented with 10% (*v/v*) fetal bovine serum (Gibco, Waltham, MA, USA), whereas the Balb/3T3 cells were grown in Dulbecco’s Modified Eagle’s Medium (Sigma-Aldrich) supplemented with 10% (*v/v*) heat-inactivated fetal bovine serum, glutamine (0.584 g/L), and 10 mL/L 100× penicillin-streptomycin (Gibco).

The essential oils or pure compounds identified from these oils were dissolved in dimethyl sulfoxide (DMSO) and serially diluted with growth medium in white 96-well microtiter plates. 20,000 bloodstream forms of *T. brucei* or Balb/3T3 cells were added to each well in a final volume of 200 μL. In the case of mammalian cells, we also tested 2000 cells/well with similar results. To avoid any damage to the cells, the concentration of DMSO in the solution was never higher than 1% (no cell growth inhibition was observed with this concentration of DMSO). Cell viability was verified by a drug-free control for each compound.

The plates were incubated for 48 h in the CO_2_ incubator; then 20 μL of 0.5 mM resazurine (Sigma Aldrich) was added to each well, and the plates were incubated for an additional 24 h before the fluorescence was measured with an Infinite M200 microplate reader (Tecan group, Ltd., Männedorf, Switzerland) equipped with 540 and 590 nm excitation and emission filters. The half maximal inhibitory concentration (IC_50_) values were calculated on a log inhibitor versus the response curves by non-linear regression using the GraphPad prism 5.04 software (GraphPad Software, Inc., La Jolla, CA, USA).

## 3. Results and Discussion

### 3.1. Chemical Composition of Essential Oils from Cameroonian Plants

The chemical composition of the essential oils hydrodistilled from the six Cameroonian medicinal and aromatic plants is reported in Table 1. In the essential oil obtained from neem leaves, a total of thirteen components were identified, accounting for 98.3% of the total composition. The oil was almost entirely composed by sesquiterpene hydrocarbons (97.4%), with germacrene B (74.0%) and γ-elemene (18.3%) as the predominant components. The minor constituents were (*E*)-caryophyllene (2.4%) and β-elemene (0.9%). This work represents one of the few studies on the chemical composition of neem leaf essential oils. Neem trees have been largely investigated for the oil obtained by mechanical pressure or solvent extraction from their seeds. Earlier, El-Hawary et al. [46] studied the composition of Egyptian neem leaves, reporting β-elemene (33.39%), γ-elemene (9.89%), germacrene D (9.72%), caryophyllene (6.8%), and bicyclogermacrene (5.23%) as the major compounds, while Dastan et al. [47] found γ-elemene (20.8%), germacrene B (20.3%), *trans*-caryophyllene (13.5%), hexadecanal (12.8%), and methyl linoleate (10.5%) as the major compounds in neem leaf oil from Iran.

A total of fifty-nine components were identified in the essential oil of alligator pepper, accounting for 99.4% of the total composition. The oil was dominated by oxygenated monoterpenes (83.3%), with 1,8-cineole (58.5%) and α-terpineol (19.4%) as the major compounds. Monoterpene hydrocarbons gave a minor contribution (14.9%), with β-pinene (7.1%) and α-pinene (2.0%) as the most representative compounds. Interestingly, sesquiterpenoids, which are reported as volatile marker compounds of alligator pepper, were detected in only low levels here (0.6%). The chemical composition of the *A. melegueta* seed essential oil showed a significant variability depending on the geographic origin and genetic characteristics of the samples. For example, samples from Nigeria exhibited humulene (26.23%), (*E*)-ocimene (23.22%), (*E*)-caryophyllene (19.17%), and (*S*)-2-heptyl acetate (16.22%) as the major volatile constituents [24]. On the other hand, the seeds from the Central African Republic contained high levels of β-pinene (>30%) and about 50% sesquiterpene hydrocarbons [48], and an oil sample from Cameroon was made up of β-caryophyllene (8.5%), α-humulene (31.3%), and their epoxides (17.9% and 27.7%, respectively) [49].

Fifty-seven compounds were identified in the essential oil from African cardamom leaves, accounting for 99.3% of the total composition. This oil was mainly made up of monoterpene hydrocarbons (59.8% in leaves), accompanied by lower amounts of sesquiterpene hydrocarbons (20.0%), oxygenated monoterpenes (11.0%), and oxygenated sesquiterpenes (8.4%). The major compounds were sabinene (43.9%) and (*E*)-caryophyllene (16.6%), whereas other components occurring at noteworthy levels were β-pinene (5.8%), terpinen-4-ol (3.7%), and α-pinene (2.4%). This study was the first report on the leaf essential oil from *A. daniellii*.

The essential oil extracted from the leaves of *C. anisata* was characterized by high levels of phenylpropanoids (84.0%), which were mainly represented by (*E*)-anethole (64.6%), with minor contributions by (*E*)-methyl isoeugenol (16.1%) and methyl chavicol (2.0%). Terpenoids constituted a minor part of this oil, being represented mostly by *p*-cymene (2.9%), γ-terpinene (2.4%), myrcene (2.0%), and germacrene D (2.2%).

In the essential oil of *D. cinerea* seeds, a total of forty-nine volatile components were identified, accounting for 76.0% of the oil’s composition. Oxygenated monoterpenes were the most abundant constituents (50.6%), with geraniol (18.2%), terpinen-4-ol (7.5%), linalool (4.0%), and umbellulone (3.8%) as the most representative compounds. Oxygenated sesquiterpenes gave a lower contribution (12.1%), with none of the identified components exceeding 1.8%. Among other components occurring in the oil, it is worth noting the presence of ligustrazin (5.1%), elemicin (3.0%), and decanoic acid (2.8%). To our knowledge, no previous research has reported the chemical composition of *D. cinerea* seed essential oil.

Thirty-five volatile compounds, all belonging to the sesquiterpene class (94.3%), were identified in the root essential oil from *E. giganteus* (54.7% sesquitepene hydrocarbons and 39.6% oxygenated sesquiterpenes). The major compounds were tricyclic sesquiterpenoids, which are characterized by multiple rearrangements of the caryophyllene cation [50,51], namely, silphiperfol-6-ene (23.0%), presilphiperfolan-8-ol (22.7%), presilphiperfol-7-ene (7.8%), and cameroonan-7-α-l (7.8%). Another noteworthy constituent occurring in the oil was (*E*)-caryophyllene (6.3%). The cameroonan-7-α-l, is responsible for the patchouli-like smell of the root oil [52]. The reported composition was quite consistent with that previously reported by Menut et al. [49] for root samples collected in Cameroon.

### 3.2. Inhibition of Trypanosoma brucei Proliferation

Essential oils are complex mixtures of volatile compounds with multitarget actions, the antitrypanosomal effects of which are largely unknown and barely explored. On this basis, we decided to test the in vitro inhibitory effects of a pool of essential oils taken from medicinal and aromatic plants growing in Cameroon. Some of them are known for their traditional uses in the treatment of infectious diseases and malaria [53,54] and, in the case of the Neem tree, also against *T. b. brucei* [21].

Based on the chemical analysis performed, they exhibited different chemical profiles characterized by diverse functionalized groups such as monoterpene hydrocarbons (African cardamom), oxygenated monoterpenes (*A. melegueta* and *D. cinerea*), sesquiterpene hydrocarbons (*A. indica*), sesquiterpene hydrocarbons and oxygenated sesquiterpenes (*E. giganteus*), and phenylpropanoids (*C. anisata*). In this context, the main aim of our work was to identify the chemical scaffolds of possible natural lead compounds against trypanosomiasis. 

Testing the essential oils obtained from Cameroonian plants, we obtained various degrees of inhibition on *T. brucei* proliferation, varying from not active (*A. melegueta*, *C. anisata*, and *D. cinerea*), to moderately active (*A. danielli*, *E. giganteus* and *A. indica*). Notably, the IC_50_ values on *T. brucei* were 7.65, 10.50, and 15.21 µg/mL for the essential oils from *A. danielli*, *E. giganteus*, and *A. indica*, respectively (Table 2). Furthermore, the most active oils were also evaluated for the growth inhibitory effects on Balb/3T3 cells as a reference. No effect on mammalian cells was observed with concentrations as high as 100 µg/mL, showing a noteworthy selectivity against *T. brucei* in comparison to mammalian cells, with selectivity indexes above 6.57 in all cases.

**Figure 1:**
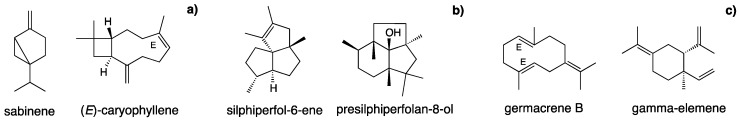
Chemical structures of the main essential oil constituents in (**a**) *Aframomum daniellii*, (**b**) *Echinops giganteus*, and (**c**) *Azadirachta indica*.

The inhibitory effects on *T. brucei* exhibited by the three essential oils highlights three classes of active compounds, i.e., monoterpene hydrocarbons (for *A. danielli*) and sesquiterpene hydrocarbons (for *A. indica* and *E. giganteus*) (Figure 1). *E giganteus* also contains high amounts of oxygenated compounds.

The toxicity of monoterpene hydrocarbons against *T. brucei* can be attributed to the high hydrophobicity of this class of compounds, which are able to easily cross the cell membrane, causing the destabilization of phospholipid bilayers and the alteration of their permeability, leading to cell damage and death [44,55]. Among these compounds, the most abundant was sabinene (43.9%) (Figure 1) which showed an IC_50_ value against *T. brucei* of 5.96 µg/mL, which is close to that of the African cardamom essential oil (7.65 µg/mL) (Table 2). Another component of this oil with detectable antitrypanosomal activity was β-pinene, showing an IC_50_ value of 11.4 µg/mL. The antitrypanosomal activity of sabinene was already reported in a previous study, although its mechanism of action on the protozoal cell has not been elucidated [56].

*E. giganteus* as well as *A. daniellii* essential oils contained the bicyclic sesquiterpene (*E*)-caryophyllene (Figure 1), which exhibited good inhibitory properties on *T. brucei* (IC_50_ value of 8.25 µg/mL). However, the content of this component is only 6.3% in *E. giganteus* and can therefore not explain the good activity of this essential oil. It is rather tricyclic sesquiterpenes that are the major constituent. This is the first report documenting the antitrypanosomal activity of the tricyclic sesquiterpenes-containing *E. giganteus* essential oil. The latter was a rich source of compounds such as silphiperfol-6-ene, presilphiperfolan-8-ol, presilphiperfol-7-ene, and cameroonan-7-α-l with an unusual skeleton (Figure 1) [26]. To date, these compounds have not been biologically investigated. Previously, the tricyclic sesquiterpene ledol was suggested to be responsible for the trypanocidal properties of *Hagenia abyssinica* (Bruce ex Steud.) J.F.Gmel. essential oil [13].

Among the other pure compounds, 1,8-cineole and terpinen-4-ol were inactive against *T. brucei* (IC_50_ > 100 µg/mL) (Table 2), and this also explained the lack of activity of the *A. melegueta* and *D. cinerea* essential oils, which are dominated by these compounds.

Finally, we demonstrated for the first time that the leaf essential oil from the neem tree, which showed an IC_50_ value of 15.21 µg/mL, can be a source of sesquiterpenes such as germacrene B and γ-elemene. Since this oil is completely dominated by sesquiterpenes (97.4%), it can be assumed that they are responsible for its antitrypanosomal activity. Further studies are needed to elucidate their mode of action and the possibility of them acting as lead compounds for the discovery of antitrypanosomal drugs.

## 4. Conclusions

In conclusion, our biological investigation into the essential oils distilled from medicinal and aromatic plants growing in Cameroon identified some terpenoids as possible lead compounds of natural antitrypanosomal drugs. Further research is encouraged to disclose their mechanisms of action and in vivo efficacy.

## Figures and Tables

**Table 1:** Chemical composition of the essential oils from *Azadirachta indica*, *Aframomum melegueta*, *Aframomum danielli*i, *Clausena anisata*, *Dichrostachys cinerea*, and *Echinops giganteus*.

No.	Component ^a^	RI calc. ^b^	RI lit. ^c^	% ^d^						ID ^f^
				*Azadirachta indica*	*Aframomum melegueta*	*Aframomum daniellii* ^e^	*Clausena anisata*	*Dichrostachys cinerea* ^e^	*Echinops giganteus* ^e^	
1	isopentyl acetate	873	869		tr ^g^					RI,MS
2	2-methyl butyl acetate	876	875		tr					RI,MS
3	2-heptanone	891	892		tr					RI,MS
4	2-heptanol	901	894		0.2					RI,MS
5	α-thujene	916	924		tr	1.0	0.1			RI,MS
6	α-pinene	921	932		2.0	2.4	0.2		tr	Std
7	α-fenchene	938	945		0.1					RI,MS
8	camphene	939	946		0.3	tr				RI,MS
9	sabinene	959	969		tr	43.9	0.6			RI,MS
10	β-pinene	963	974		7.1	5.8	0.3	0.1		Std
11	dehydro-1,8-cineole	979	988		0.1					RI,MS
12	myrcene	982	988		0.2	1.5	2.0	0.3	tr	Std
13	α-phellandrene	996	1004		0.3	tr				Std
14	δ-3-carene	1003	1008						tr	Std
15	α-terpinene	1009	1014		0.3	0.9	tr			RI,MS
16	*p*-cymene	1016	1020		1.1	1.0	2.9	0.1		Std
17	limonene	1020	1024		1.5	0.7	0.4	tr	tr	Std
18	1,8-cineole	1021	1026		58.5	0.5	0.2	2.3		Std
19	ethylhexanol	1031	1030					0.2		RI,MS
20	(*Z*)-β-ocimene	1037	1032				0.4			Std
21	(*E*)-β-ocimene	1041	1044		0.1	0.3	0.3			RI,MS
22	γ-terpinene	1050	1054		0.9	1.9	2.4			Std
23	*cis*-sabinene hydrate	1057	1065			1.1				RI,MS
24	*cis*-linalool oxide	1071	1067				tr	0.1		RI,MS
25	terpinolene	1079	1086		0.8	0.4	0.1		tr	Std
26	*p*-cymenene	1086	1089		0.2					RI,MS
27	ligustrazin	1081	1083					5.1		RI,MS
28	*trans*-sabinene hydrate	1089	1098			0.9				RI,MS
29	2-nonanone	1094	1094		tr					RI,MS
30	linalool	1096	1095		tr	1.8	tr	4.0		Std
31	*n*-nonanal	1105	1100		tr					RI,MS
32	*endo*-fenchol	1108	1114		0.3					RI,MS
33	*cis*-*p*-menth-2-en-1-ol	1113	1118			0.2		0.3		RI,MS
34	α-campholenal	1123	1122		tr			0.1		RI,MS
35	*trans*-pinocarveol	1128	1135		0.2			0.7		Std
36	*trans*-*p*-menth-2-en-1-ol	1131	1136			0.1		0.3		RI,MS
37	*cis*-β-terpineol	1142	1140		tr					RI,MS
38	*cis*-verbenol	1142	1137					0.2		RI,MS
39	*trans*-pinocamphone	1151	1158					0.4		RI,MS
40	pinocarvone	1152	1160		tr			0.1		RI,MS
41	borneol	1156	1165		0.2			0.7		Std
42	*p*-mentha-1,5-dien-8-ol	1158	1166		1.1					RI,MS
43	*cis*-pinocamphone	1162	1172					0.8		RI,MS
44	umbelullone	1166	1167					3.8		RI,MS
45	terpinen-4-ol	1167	1174		1.4	3.7	tr	7.5		Std
46	*cis*-pinocarveol	1175	1182		tr					RI,MS
47	cryptone	1183	1183		tr					RI,MS
48	*p*-cymen-8-ol	1178	1179		tr		tr	0.3		RI,MS
49	α-terpineol	1181	1186		19.4	0.2	tr	3.3		Std
50	myrtenal	1184	1195		0.2	0.1		0.2		Std
51	myrtenol	1186	1194		0.2	0.4		1.1		Std
52	*cis*-piperitol	1199	1195			tr				RI,MS
53	γ-terpineol	1195	1199		tr					RI,MS
54	methyl chavicol	1196	1195				2.0			RI,MS
55	*trans*-piperitol	1205	1207					0.1		RI,MS
56	*trans*-carveol	1217	1215		tr			0.2		RI,MS
57	*cis*-carveol	1228	1226		tr					RI,MS
58	thymol methyl ether	1224	1232		tr					RI,MS
59	nerol	1229	1227					0.2		Std
60	citronellol	1231	1223					0.3		Std
61	carvone	1240	1239		tr					Std
62	carvacrol methyl ether	1237	1241		tr					RI,MS
63	neral	1241	1235					0.2		Std
64	piperitone	1250	1249					0.3		RI,MS
65	(*Z*)-anethole	1250	1249				0.3			RI,MS
66	*p*-anisaldehyde	1251	1247				0.7			RI,MS
67	geraniol	1251	1249					18.2		Std
68	*trans*-ascaridol glycol	1262	1266			tr				RI,MS
69	(*E*)-cinnamaldehyde	1267	1267		tr					RI,MS
70	phellandral	1269	1273		0.1					RI,MS
71	isobornyl acetate	1276	1283			tr				Std
72	(*E*)-anethole	1287	1282				64.6			Std
73	thymol	1291	1289		0.1			0.9		Std
74	*trans*-sabinyl acetate	1291	1289			tr				RI,MS
75	methyl myrtenate	1292	1293					2.0		RI,MS
76	carvacrol	1301	1298		0.5		tr	0.7		Std
77	*cis*-pinocarvyl acetate	1303	1311			0.1				RI,MS
78	myrtenyl acetate	1316	1324			1.9				RI,MS
79	silphiperfol-5-ene	1318	1326						2.1	RI,MS
80	δ-elemene	1326	1335	0.1			0.1			RI,MS
81	presilphiperfol-7-ene	1328	1334						7.8	RI,MS
82	silphinene	1333	1340						1.7	RI,MS
83	7-*epi*-silphiperfol-5-ene	1336	1349						3.5	RI,MS
84	α-terpinyl acetate	1341	1346		tr			0.3		RI,MS
85	α-copaene	1362	1374	0.2		0.2	tr			Std
86	β-bourbonene	1369	1387	0.3		tr				RI,MS
87	modheph-2-ene	1362	1382						3.0	RI,MS
88	silphiperfol-6-ene	1373	1377						23.0	RI,MS
89	β-bourbonene	1377	1387				0.1			RI,MS
90	β-cubebene	1377	1387			tr				RI,MS
91	α-isocomene	1379	1387						2.4	RI,MS
92	β-elemene	1380	1389	0.9		tr	0.1			Std
93	decanoic acid	1380	1386					2.8		RI,MS
94	anisyl methyl ketone	1382	1380				0.1			RI,MS
95	*iso*-longifolene	1383	1389						tr	RI,MS
96	β-isocomene	1400	1407						2.1	RI,MS
97	α-gurjunene	1400	1409						tr	Std
98	(*E*)-caryophyllene	1402	1417	2.4	tr	16.6	0.8		6.3	Std
99	methyl eugenol	1406	1403				0.3			RI,MS
100	α-*trans*-bergamotene	1425	1432			0.1				RI,MS
101	isoamyl benzoate	1433	1433		tr					RI,MS
102	γ-elemene	1427	1434	18.3			0.1			RI,MS
103	α-humulene	1436	1452	0.4	tr	1.5	0.8		2.0	Std
104	geranyl acetone	1449	1453					1.2		RI,MS
105	(*E*)-β-farnesene	1450	1454			tr			tr	RI,MS
106	germacrene D	1465	1484	0.5		0.3	2.2		0.3	RI,MS
107	selina-4,11-diene	1467	1474			0.1				RI,MS
108	β-selinene	1469	1489		tr					RI,MS
109	*ar*-curcumene	1472	1479				tr		0.1	RI,MS
110	bicyclogermacrene	1480	1500			0.1	0.1			RI,MS
111	benzaldehyde, 3,4-dimethoxy-	1482	1489				0.2			RI,MS
112	(*E*)-β-ionone	1481	1487	0.5						Std
113	*epi*-cubebol	1489	1493						0.1	RI,MS
114	α-zingiberene	1492	1493				0.1			RI,MS
115	(*Z*)-α-bisabolene	1493	1506			0.1				RI,MS
116	silphiperfolan-6-α-ol	1496	1507						1.0	RI,MS
117	β-bisabolene	1498	1505			0.9				RI,MS
118	(*E*)-methyl isoeugenol	1499	1491				16.1			RI,MS
119	cameroonan-7-α-ol	1500	1510						7.1	RI,MS
120	β-bisabolene	1506	1505				0.3			RI,MS
121	7-*epi*-α-selinene	1507	1520		tr					RI,MS
122	(*E*,*E*)-α-farnesene	1508	1505				0.3			Std
123	*trans*-calamenene	1508	1521			tr				RI,MS
124	*δ*-cadinene	1510	1522	0.2		tr	0.1		0.3	RI,MS
125	silphiperfolan-7-β-ol	1510	1519						2.5	RI,MS
126	β-sesquiphellandrene	1520	1521				tr			RI,MS
127	selina-3,7(11)-diene	1531	1545	0.2						RI,MS
128	silphiperfolan-6-β-ol	1535	1546						1.7	RI,MS
129	hedycaryol	1536	1546			1.5				RI,MS
130	germacrene B	1546	1559	74.0			0.3			RI,MS
131	elemicin	1556	1555					3.0		RI,MS
132	(*E*)-nerolidol	1556	1561			0.7				Std
133	isoaromadendrene epoxide	1560	1572					1.8		RI,MS
134	prenopsan-8-ol	1564	1575						3.2	RI,MS
135	caryophyllene oxide	1564	1582			2.2		1.1		Std
136	(3*Z*)-hexenyl benzoate	1566	1565	0.3						RI,MS
137	spathulenol	1568	1576				0.1			RI,MS
138	presilphiperfolan-8-ol	1578	1585						22.7	MS
139	guaiol	1583	1600			0.5				RI,MS
140	humulene epoxide II	1590	1608			0.1	tr			RI,MS
141	10-*epi*-γ-eudesmol	1600	1622		tr	0.3				RI,MS
142	eremoligenol	1611	1629			0.4				RI,MS
143	γ-eudesmol	1615	1630		tr	0.4				RI,MS
144	1,10-di-*epi*-cubenol	1619	1618						0.1	RI,MS
145	β-eudesmol	1631	1649		0.2	1.5				RI,MS
146	α -acorenol	1628	1632					1.0		RI,MS
147	caryophylla-4(12),8(13)-dien-5-ol ^h^	1630	1639			0.2			tr	RI,MS
148	*epi*-α -muurolol	1635	1640				tr	0.7	0.4	RI,MS
149	α -muurolol	1640	1644						0.1	RI,MS
150	α -eudesmol	1636	1652			0.5				RI,MS
151	intermedeol	1639	1666			0.1				RI,MS
152	α-cadinol	1647	1652				tr	1.4	0.4	RI,MS
153	ageratochromene	1655	1658					0.8		RI,MS
154	α-bisabolol	1673	1685		tr	0.4				Std
155	3-oxo-β-ionone	1678	1685					0.9		RI,MS
156	cyperotundone	1688	1695					0.9		RI,MS
157	(2*E*-6*Z*)-farnesol	1709	1698					2.4		RI,MS
158	curcuphenol	1716	1717						0.4	RI,MS
159	(2*E*-6*Z*)-farnesal	1718	1713					1.0		RI,MS
160	(2*E*-6*E*)-farnesal	1737	1740					1.7		RI,MS
161	*n*-tricosane	2300	2300		0.1					Std
	Oil yield (%)			0.01	0.3	0.2	2.0	0.4	1.8	
	Total identified (%)			98.3	99.4	99.3	99.6	76.0	94.3	
	Grouped compounds (%)									
	Monoterpene hydrocarbons				14.9	59.8	0.6	0.6	tr	
	Oxygenated monoterpenes				83.3	11.0	0.3	50.6		
	Sesquiterpene hydrocarbons			97.4	0.2	20.0	5.2		54.7	
	Oxygenated sesquiterpenes				0.4	8.4	0.2	12.1	39.6	
	Phenylpropanoids						84.0			
	Others			0.9	0.6		0.2	12.7		

^a^ Components are reported in order of their elution from an HP-5MS capillary column; ^b^ Retention index (RI) experimentally determined using a mixture of C_8_-C_30_ of n-alkanes; ^c^ Retention index taken from ADAMS [41] and/or NIST 08 [42] for an apolar capillary column; ^d^ Relative percentage values are means of three determinations with a Relative Standard Deviation (RSD%) below 15% for the most abundant components; ^e^ Analytical data are taken from Pavela et al. [26]; ^f^ Identification methods: standard (std), based on the comparison of RT (retention time), RI, and MS (mass spectronomy) with authentic compounds; RI, based on correspondence of calculated RI with those reported in ADAMS and NIST 08; MS, based on comparison with the WILEY, ADAMS, FFNSC2, and NIST 08 MS databases; ^g^ traces, % <0.1; ^h^ Correct isomer not identified. ID: Identity.

**Table 2:** Inhibitory effects of essential oils from Cameroonian plants against *Trypanosoma brucei brucei* TC221 and Balb/3T3 cells.

Treatment	IC_50_ (µg/mL)		Selectivity Index (SI)
	*T. b. brucei* (TC221)	Balb/3T3	
**Essential oils**			
*Aframomum danielli*	7.65 ± 1.1	>100	>13.1
*Dichrostachys cinerea*	>100	-	-
*Echinops giganteus*	10.50 ± 1.7	>100	>9.52
*Azadirachta indica*	15.21 ± 0.97	>100	>6.57
*Aframomum melegueta*	>100	-	-
*Clausena anisata*	>100	-	-
**Pure compounds**	**µg/mL (µM)**	**µg/mL (µM)**	
Sabinene	5.96 ± 1.3 (43.8)	>100	>16.7
β-Pinene	11.4 ± 2.6 (83.7)	>100	>8.77
1,8-Cineole	>100	-	
Terpinen-4-ol	>100	-	
(*E*)-Caryophyllene	8.25 ± 1.3 (40.4)	>100	>12.1
**Reference drug**	**µg/mL (µM)**	**µg/mL (µM)**	
Suramin	0.0286 ± 0.0008 (0.0220)	-	

IC**_50_:** half maximal inhibitory concentration.
